# Peroxisome proliferator-activated receptor delta +294T > C polymorphism and serum lipid levels in the Guangxi Bai Ku Yao and Han populations

**DOI:** 10.1186/1476-511X-9-145

**Published:** 2010-12-21

**Authors:** Lin Miao, Rui-Xing Yin, Dong-Feng Wu, Xiao-Li Cao, Qing Li, Xi-Jiang Hu, Ting-Ting Yan, Lynn Htet Htet Aung, De-Zhai Yang, Wei-Xiong Lin

**Affiliations:** 1 Department of Cardiology, Institute of Cardiovascular Diseases, the First Affiliated Hospital, Guangxi Medical University, 22 Shuangyong Road, Nanning 530021, Guangxi, People's Republic of China; 2 Department of Molecular Biology, Medical Scientific Research Center, Guangxi Medical University, 22 Shuangyong Road, Nanning 530021, Guangxi, People's Republic of China

## Abstract

**Background:**

The association of peroxisome proliferator-activated receptor delta (PPARD) +294T > C polymorphism and serum lipid levels is inconsistent in several previous studies. Bai Ku Yao is an isolated subgroup of the Yao minority in China. The present study was undertaken to detect the association of PPARD +294T > C (rs2016520) polymorphism and several environmental factors with serum lipid levels in the Guangxi Bai Ku Yao and Han populations.

**Methods:**

A total of 609 subjects of Bai Ku Yao and 573 participants of Han Chinese were randomly selected from our previous stratified randomized cluster samples. Genotyping of the PPARD +294T > C polymorphism was performed by polymerase chain reaction and restriction fragment length polymorphism combined with gel electrophoresis, and then confirmed by direct sequencing.

**Results:**

The levels of serum total cholesterol (TC), high-density lipoprotein cholesterol (HDL-C), apolipoprotein (Apo) AI and ApoB were lower in Bai Ku Yao than in Han (*P *< 0.001 for all). The frequency of T and C alleles was 77.50% and 22.50% in Bai Ku Yao, and 72.43% and 27.57% in Han (*P *< 0.01); respectively. The frequency of TT, TC and CC genotypes was 60.59%, 33.83% and 5.53% in Bai Ku Yao, and 52.18%, 40.50% and 7.32% in Han (*P *< 0.05); respectively. The subjects with CC genotype in Bai Ku Yao had higher serum LDL-C and ApoB levels and lower the ratio of ApoAI to ApoB than the subjects with TT and TC genotypes in females but not in males. The C allele carriers in Han had higher serum TC levels in males (*P *< 0.01) and ApoB levels in females (*P *< 0.05) than the C allele noncarriers. Serum TC and ApoB levels were correlated with genotypes in Han (*P *< 0.05 for each) but not in Bai Ku Yao. Serum lipid parameters were also correlated with sex, age, body mass index, alcohol consumption, cigarette smoking, and blood pressure in both ethnic groups.

**Conclusions:**

These results suggest that the association of PPARD +294T > C polymorphism and serum lipid levels is different between the Bai Ku Yao and Han populations. The discrepancy between the two ethnic groups might partly result from different PPARD +294T > C polymorphism or PPARD gene-enviromental interactions.

## Introduction

Elevated levels of total cholesterol (TC), triglyceride (TG), low-density lipoprotein cholesterol (LDL-C) and apolipoprotein (Apo) B, together with decreased levels of high-density lipoprotein cholesterol (HDL-C), and ApoAI, are associated with an increased risk of coronary artery disease (CAD) [1-4]. Genetic and environmental factors [4-10] and their interactions [11,12] are believed to regulate both metabolic and cellular function, and the serum lipid concentrations. Twin and family studies have shown the variable estimates of heritability for plasma lipid phenotypes ranging from 40 to 60% in different studies supporting the idea of a strong genetic component in the development of dyslipidemia [13-17].

Peroxisome proliferator-activated receptors (PPARs) are nuclear transcription factors involved in the regulation of lipid and glucose metabolism. The family includes PPAR-alpha (PPARA), PPAR-gamma (PPARG), and PPAR-delta (PPARD). PPARA is mainly expressed in liver, muscle, kidney and heart, PPARG is most abundant in adipocytes, intestinal cells and macrophages and PPARD is expressed in many tissues [18-20]. Each subgroup is activated by a certain variety of fatty acids and their derivatives and by specific pharmacological ligands. After forming obligate heterodimers with the retinoid × receptor, PPARs bind to specific elements in the promoter region of target genes, thereby altering metabolism by activating a network of downstream genes [21,22]. PPARs are encoded by separate genes and characterized by distinct tissue and developmental distribution patterns. The PPARD gene, located on chromosome 6p21.2-p21.1, is involved in cellular transport, storage and metabolism of lipids [23], which ultimately translates into a metabolic response. This gene is equally actively expressed in both adipose tissue and skeletal muscles, primarily in slow twitch muscle fibers. Treatment of obese rhesus monkeys with the synthetic PPARD agonist GW501516 resulted in an increase of HDL-C levels and a decrease of plasma TG [24,25]. In addition, db/db mice expressing an activated form of PPARD are resistant to obesity and hyperlipidemia when overfed and muscle-specific overexpression of the receptor increased the number of muscle fibers with high oxidative metabolic capability [26,27]. These results suggest that PPARD may play a role in the development of metabolic perturbations associated with dyslipidemia and predisposing to atherosclerosis. The +294T > C (rs2016520) polymorphism in the 5'-untranslated region in exon 4 of the PPARD gene is located 87 nucleotides upstream of the start codon. It was shown that the single nucleotide polymorphism (SNP) influenced binding of Sp-1 resulting in higher transcriptional activity for the rare C allele than the common T allele [28]. Several previous studies have showed that the PPARD +294T > C polymorphism was associated with modifications of serum lipid concentrations in healthy subjects and the risk of CAD [28-35] in dyslipidemic women and hypercholesterolemic men and cholesterol metabolites in Alzheimer's disease patients [36]. But the results are inconsistent in diverse populations [34,37].

There are 56 ethnic groups in China. Han is the largest ethnic group and Yao is the eleventh largest minority among the 55 minority groups according to the population size. Bai Ku Yao (White-trouser Yao), an isolated branch of the Yao minority, is named so because all of men wear white knee-length knickerbockers. The population size is about 30000. Because of isolation from the other ethnic groups, the special customs and cultures including their clothing, intra-ethnic marriages, ballad, funeral, bronze drum, spinning top, dietary habits, and corn wine and rum intakes are still completely preserved to the present day. In previous epidemiological studies, we showed that several serum lipid parameters were lower in Bai Ku Yao than in Han Chinese from the same area [6,7]. These differences in serum lipid profiles are still not well known. We hypothesized that the genetic and environmental risk factors for dyslipidemia may be different between the two ethnic groups. Therefore, the aim of the present study was to detect the association of PPARD +294T > C polymorphism and several environmental factors with serum lipid parameters in the Guangxi Bai Ku Yao and Han populations.

## Materials and methods

### Study population

A total of 609 participants of Bai Ku Yao who reside in Lihu and Baxu villages in Nandan County, Guangxi Zhuang Autonomous Region, People's Republic of China were randomly selected from our previous stratified randomized cluster samples [6,7]. The ages of the subjects ranged from 15 to 80 years, with an average age of 41.75 ± 15.46 years. There were 288 males (47.29%) and 321 females (52.71%). All subjects were rural agricultural workers. The subjects accounted for 2.03% of total Bai Ku Yao population. During the same period, a total of 573 subjects of Han Chinese who reside in the same villages were also randomly selected from our previous stratified randomized cluster samples [6,7]. The average age of the subjects was 42.18 ± 15.63 years (range 15 to 80). There were 260 men (45.38%) and 313 women (54.62%). All of them were also rural agricultural workers. All study subjects were essentially healthy and had no evidence of any chronic illness, including hepatic, renal, or thyroid. The participants with a history of heart attack or myocardial infarction, stroke, congestive heart failure, diabetes or fasting blood glucose ≥ 7.0 mmol/L determined by glucose meter have been excluded. The participants were not taking medications known to affect serum lipid levels (lipid-lowering drugs such as statins or fibrates, beta-blockers, diuretics, or hormones). The present study was approved by the Ethics Committee of the First Affiliated Hospital, Guangxi Medical University. Informed consent was obtained from all subjects after they received a full explanation of the study.

### Epidemiological survey

The survey was carried out using internationally standardized methods, following a common protocol [38]. Information on demographics, socioeconomic status, and lifestyle factors was collected with standardized questionnaires. The alcohol information included questions about the number of liangs (about 50 g) of rice wine, corn wine, rum, beer, or liquor consumed during the preceding 12 months. Alcohol consumption was categorized into groups of grams of alcohol per day: < 25 and ≥ 25. Smoking status was categorized into groups of cigarettes per day: < 20 and ≥ 20. At the physical examination, several anthropometric parameters, such as height, weight, and waist circumference were measured. Sitting blood pressure was measured three times with the use of a mercury sphygmomanometer after the subjects had a 5-minute rest, and the average of the three measurements was used for the level of blood pressure. Systolic blood pressure was determined by the first Korotkoff sound, and diastolic blood pressure by the fifth Korotkoff sound. Body weight, to the nearest 50 grams, was measured using a portable balance scale. Subjects were weighed without shoes and in a minimum of clothing. Height was measured, to the nearest 0.5 cm, using a portable steel measuring device. From these two measurements body mass index (BMI, kg/m^2^) was calculated.

### Biochemical analysis

A venous blood sample of 8 mL was obtained from all subjects between 8 and 11 AM, after at least 12 hours of fasting, from a forearm vein after venous occlusion for few seconds in a sitting position. A part of the sample (3 mL) was collected into glass tubes and allowed to clot at room temperature, and used to determine serum lipid levels. Another part of the sample (5 mL) was transferred to tubes with anticoagulate solution (4.80 g/L citric acid, 14.70 g/L glucose, and 13.20 g/L tri-sodium citrate) and used to extract DNA. Immediately following clotting serum was separated by centrifugation for 15 minutes at 3000 rpm. The levels of TC, TG, HDL-C, and LDL-C in samples were determined by enzymatic methods with commercially available kits, Tcho-1, TG-LH (RANDOX Laboratories Ltd., Ardmore, Diamond Road, Crumlin Co. Antrim, United Kingdom, BT29 4QY), Cholestest N HDL, and Cholestest LDL (Daiichi Pure Chemicals Co., Ltd., Tokyo, Japan); respectively. Serum ApoAI and ApoB levels were detected by the immunoturbidimetric immunoassay using a commercial kit (RANDOX Laboratories Ltd.). All determinations were performed with an autoanalyzer (Type 7170A; Hitachi Ltd., Tokyo, Japan) in the Clinical Science Experiment Center of the First Affiliated Hospital, Guangxi Medical University [6,7].

### DNA amplification and genotyping

Genomic DNA was isolated from peripheral blood leukocytes using the phenol-chloroform method [8-12]. The extracted DNA was stored at 4°C until analysis. Genotyping of the PPARD +294T > C polymorphism was performed by polymerase chain reaction and restriction fragment length polymorphism (PCR-RFLP) [35,37]. PCR amplification was performed using 5'-CATGGTATAGCACTGCAGGAA-3' and 5'-CTTCCTCCTGTGGCTGCTC-3' (Sangon, Shanghai, People's Republic of China) as the forward and reverse primer pairs; respectively. Each amplification reaction was performed using 100 ng genomic DNA in 25 μL of reaction mixture consisting of 1.0 μL of each primer (10 μmol/L), 12.5 μL 2 × Taq PCR MasterMix (constituent: 0.1 U *Taq *polymerase/μL, 500 μM dNTP each and PCR buffer). After initial denaturizing at 95°C for 5 min, the reaction mixture was subjected to 30 cycles of 45 s denaturation at 94°C, 45 s annealing at 62°C and extension 45 s at 72°C, followed by a final 8 min extension at 72°C. After electrophoresis on a 2.0% agarose gel with 0.5 μg/mL ethidium bromide, the amplification products were visualized under ultraviolet light. Then 5 U of *Bsl*I restriction enzyme was added directly to the PCR products (6 μL) and digested at 55°C overnight. After restriction enzyme digestion of the amplified DNA, the genotypes were identified by electrophoresis on 2.5% agarose gels and visualized with ethidium-bromide staining ultraviolet illumination. The genotypes were scored by an experienced reader blinded to epidemiological data and serum lipid levels. Six samples (TT, TC and CC genotypes in two; respectively) detected by the PCR-RFLP were also confirmed by direct sequencing. The PCR products were purified by low melting point gel electrophoresis and phenol extraction, and then the DNA sequences were analyzed in Shanghai Sangon Biological Engineering Technology & Services Co., Ltd., People's Republic of China.

### Diagnostic criteria

The normal values of serum TC, TG, HDL-C, LDL-C, ApoAI, ApoB levels, and the ratio of ApoAI to ApoB in our Clinical Science Experiment Center were 3.10-5.17, 0.56-1.70, 0.91-1.81, 2.70-3.20 mmol/L, 1.00-1.78, 0.63-1.14 g/L, and 1.00-2.50; respectively. The individuals with TC > 5.17 mmol/L and/or TG > 1.70 mmol/L were defined as hyperlipidemic [6,7]. Hypertension was diagnosed according to the criteria of 1999 World Health Organization-International Society of Hypertension Guidelines for the management of hypertension [39,40]. The diagnostic criteria of overweight and obesity were according to the Cooperative Meta-analysis Group of China Obesity Task Force. Normal weight, overweight and obesity were defined as a BMI < 24, 24-28, and > 28 kg/m^2^; respectively [41].

### Statistical analyses

Epidemiological data were recorded on a pre-designed form and managed with Excel software. All statistical analyses were done with the statistical software package SPSS 13.0 (SPSS Inc., Chicago, Illinois). Quantitative variables were expressed as mean ± standard deviation (serum TG levels were presented as medians and interquartile ranges). Qualitative variables were expressed as percentages. Allele frequency was determined via direct counting, and the standard goodness-of-fit test was used to test the Hardy-Weinberg equilibrium. Difference in genotype distribution between the groups was obtained using the chi-square test. The difference in general characteristics between Bai Ku Yao and Han was tested by the Student's unpaired *t*-test. The association of genotypes and serum lipid parameters was tested by analysis of covariance (ANCOVA). Sex, age, BMI, blood pressure, alcohol consumption, cigarette smoking were adjusted for the statistical analysis. In order to evaluate the association of serum lipid levels with genotypes (TT = 1, TC = 2, CC = 3) and several environment factors, multiple linear regression analysis with stepwise modeling was also performed in the combined population of Bai Ku Yao and Han, Bai Ku Yao, Han; respectively. A *P *value of less than 0.05 was considered statistically significant.

## Results

### General characteristics and serum lipid levels

Table 1 gives the general characteristics and serum lipid levels between the Bai Ku Yao and Han populations. The levels of height, weight, BMI, serum TC, HDL-C, ApoAI and ApoB were lower in Bai Ku Yao than in Han Chinese (*P *< 0.05-0.001), whereas the ratio of ApoAI to ApoB was higher in Bai Ku Yao than in Han (*P *< 0.05). There were no significant differences in the levels of systolic blood pressure, diastolic blood pressure, pulse pressure, serum TG, LDL-C, age structure, the percentages of subjects who consumed alcohol or smoked cigarettes, or the ratio of male to female between the two ethnic groups (*P *> 0.05 for all).

**Table 1:** Comparison of demographic, lifestyle characteristics and serum lipid levels between Bai Ku Yao and Han Chinese

Parameter	Bai Ku Yao	Han Chinese	*t *(χ)^2^	*P*
Number	609	573	-	-
Male/female	288/321	260/313	0.436	0.509
Age (years)	41.75 ± 15.46	42.18 ± 15.63	-0.480	0.631
Height (cm)	152.56 ± 7.40	154.33 ± 8.40	-3.828	0.000
Weight (kg)	51.65 ± 7.39	53.62 ± 8.92	-4.151	0.000
Body mass index (kg/m^2^)	22.14 ± 2.41	22.48 ± 3.03	-2.109	0.035
Systolic blood pressure (mmHg)	119.71 ± 17.96	120.73 ± 16.00	-1.036	0.300
Diastolic blood pressure (mmHg)	75.80 ± 9.64	75.97 ± 10.40	-0.293	0.769
Pulse pressure (mmHg)	43.91 ± 13.24	44.76 ± 11.00	-1.217	0.224
Cigarette smoking [n (%)]				
Nonsmoker	423 (69.5)	404 (70.5)		
< 20 cigarettes/day	82 (13.4)	72 (12.6)		
≥ 20 cigarettes/day	104 (17.1)	97 (16.9)	0.233	0.890
Alcohol consumption [n (%)]				
Nondrinker	343 (56.3)	342 (59.7)		
< 25 g/day	163 (26.8)	149 (26.0)		
≥ 25 g/day	103 (16.9)	82 (14.3)	1.919	0.383
Total cholesterol (mmol/L)	4.35 ± 0.92	4.76 ± 1.03	-7.215	0.000
Triglycerides (mmol/L)	0.98 (0.78)	1.01 (0.66)	-0.956	0.339
HDL-C (mmol/L)	1.68 ± 0.41	1.92 ± 0.49	-8.968	0.000
LDL-C (mmol/L)	2.57 ± 0.77	2.61 ± 0.76	-0.823	0.411
Apolipoprotein (Apo) AI (g/L)	1.32 ± 0.31	1.41 ± 0.24	-5.896	0.000
ApoB (g/L)	0.83 ± 0.23	0.91 ± 0.22	-5.771	0.000
ApoAI/ApoB	1.71 ± 0.72	1.64 ± 0.49	1.998	0.046

HDL-C, high-density lipoprotein cholesterol; LDL-C, low-density lipoprotein cholesterol. The value of TG was presented as median (interquartile range). The difference between the two ethnic groups was determined by the Wilcoxon-Mann-Whitney test.

### Results of electrophoresis and genotyping

**Figure 1:**
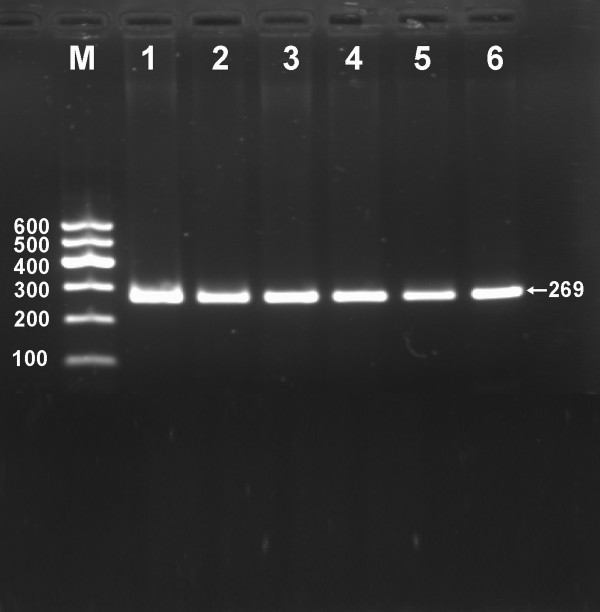
**Electrophoresis of PCR products of the samples**. Lane M, 100 bp marker ladder; lanes 1-6, samples. The 269 bp bands are the target genes.

**Figure 2:**
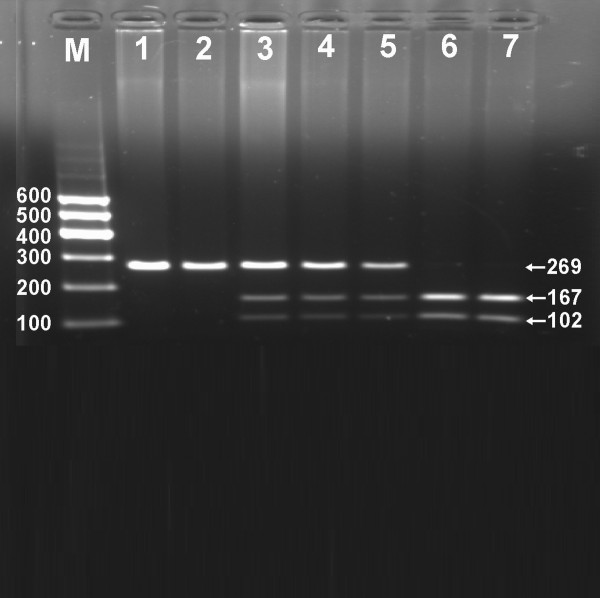
**Genotyping of the PPARD +294T > C polymorphism**. Lane M, 100 bp marker ladder; lanes 1 and 2, TT genotype (269 bp); lanes 3-5, TC genotype (269-, 167- and 102-bp); and lanes 6 and 7, CC genotype (167- and 102-bp).

After the genomic DNA of the samples was amplified by PCR and imaged by 2.0% agarose gel electrophoresis, the purpose gene of 269 bp nucleotide sequences could be found in all samples (Figure 1). The genotypes identified were named according to the presence or absence of the enzyme restriction sites, when a T to C transversion at +294 locus of the PPARD gene. The presence of the cutting site indicates the C allele, while its absence indicates the T allele (cannot be cut). Thus, the TT genotype is homozygote for the absence of the site (band at 269 bp), TC genotype is heterozygote for the absence and presence of the site (bands at 269-, 167- and 102-bp), and CC genotype is homozygote for the presence of the site (bands at 167- and 102- bp; Figure 2). The genotype distribution was consistent with the Hardy-Weinberg equilibrium.

### Genotypic and allelic frequencies

The genotypic and allelic frequencies of PPARD +294T > C polymorphism are shown in Table 2. The frequency of T and C alleles was 77.50% and 22.50% in Bai Ku Yao, and 72.43% and 27.57% in Han (*P *< 0.01); respectively. The frequency of TT, TC and CC genotypes was 60.59%, 33.83% and 5.53% in Bai Ku Yao, and 52.18%, 40.50% and 7.32% in Han (*P *< 0.05); respectively. There was no significant difference in the genotypic and allelic frequencies between males and females in both ethnic groups.

**Table 2:** Comparison of the genotype and allele frequencies of PPARD +294T > C polymorphism in Bai Ku Yao and Han Chinese [n (%)]

Group	n	Genotype			Allele	
		
		TT	TC	CC	T	C
Bai Ku Yao	609	369 (60.59)	206 (33.83)	34 (5.53)	944 (77.50)	274 (22.50)
Han Chinese	573	299 (52.18)	232 (40.50)	42 (7.32)	830 (72.43)	316 (27.57)
χ^2^	-	8.632			8.130	
*P*	-	0.013			0.004	
Bai Ku Yao						
Male	288	183 (63.54)	93 (32.30)	12 (4.16)	459 (79.69)	117 (20.31)
Female	321	186 (57.95)	113 (35.20)	22 (6.85)	486 (75.47)	158 (24.53)
χ^2^	-	3.128			3.104	
*P*	-	0.209			0.078	
Han Chinese						
Male	260	146 (56.15)	97 (37.31)	17 (6.54)	392 (74.81)	132 (25.19)
Female	313	153 (48.88)	135 (43.13)	25 (7.99)	441 (70.45)	185 (29.55)
χ^2^	-	3.036			2.718	
*P*	-	0.219			0.099	

### Results of sequencing

**Figure 3:**
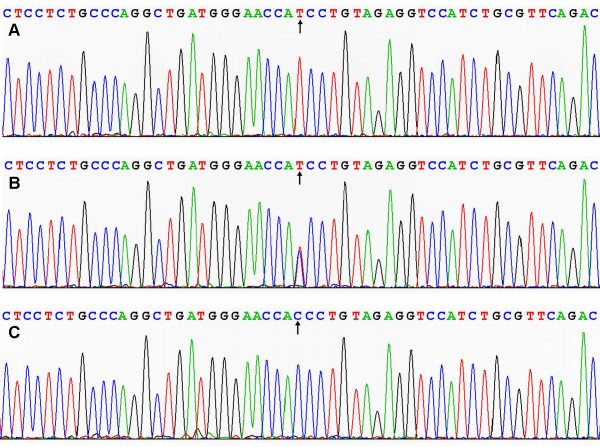
**A part of the nucleotide sequence of PPARD +294T > C polymorphism**. (A) TT genotype; (B) TC genotype; (C) CC genotype.

The results were shown as TT, TC and CC genotypes by PCR-RFLP, the TT, TC and CC genotypes were also confirmed by sequencing (Figure 3); respectively.

### Genotypes and serum lipid levels

As shown in Table 3, the levels of LDL-C, ApoB and the ratio of ApoAI to ApoB in Bai Ku Yao were significant difference among the three genotypes in females but not in males (*P *< 0.05 for all). The subjects with CC genotype had higher serum LDL-C and ApoB levels and lower the ratio of ApoAI to ApoB than the subjects with TT and TC genotypes in females.

**Table 3:** Genotypic frequencies of the PPARD +294T > C polymorphism and serum lipid levels between the Bai Ku Yao and Han populations

Genotype	n	TC (mmol/L)	TG (mmol/L)	HDL-C (mmol/L)	LDL-C (mmol/L)	ApoAI (g/L)	ApoB (g/L)	ApoAI/ (g/L)
Bai Ku Yao								
TT	369	4.35 ± 0.97	1.00(0.66)	1.67 ± 0.42	2.59 ± 0.81	1.31 ± 0.32	0.84 ± 0.22	1.69 ± 0.73
TC	206	4.29 ± 0.79	0.98(0.63)	1.69 ± 0.39	2.50 ± 0.68	1.32 ± 0.28	0.82 ± 0.24	1.74 ± 0.69
CC	34	4.63 ± 0.96	1.92(0.79)	1.72 ± 0.51	2.79 ± 0.83	1.36 ± 0.38	0.89 ± 0.25	1.68 ± 0.85
*F*	-	1.951	0.102	0.339	2.454	0.286	1.658	0.376
*P*	-	0.143	0.950	0.713	0.087	0.751	0.191	0.687
TT	369	4.35 ± 0.97	1.00(0.66)	1.67 ± 0.42	2.59 ± 0.81	1.31 ± 0.32	0.84 ± 0.22	1.69 ± 0.73
TC/CC	240	4.34 ± 0.82	0.97(0.73)	1.69 ± 0.41	2.53 ± 0.71	1.32 ± 0.30	0.83 ± 0.24	1.74 ± 0.72
*F*	-	0.021	0.319	0.566	0.604	0.082	0.172	0.495
*P*	-	0.884	0.749	0.452	0.437	0.775	0.679	0.482
Male								
TT	183	4.35 ± 1.14	1.03(0.74)	1.69 ± 0.48	2.54 ± 0.96	1.35 ± 0.37	0.81 ± 0.23	1.83 ± 0.91
TC	93	4.38 ± 0.89	1.21(1.01)	1.73 ± 0.46	2.48 ± 0.79	1.38 ± 0.33	0.82 ± 0.23	1.84 ± 0.84
CC	12	4.73 ± 1.07	1.53(1.15)	1.84 ± 0.50	2.65 ± 1.01	1.52 ± 0.41	0.82 ± 0.24	2.16 ± 1.20
*F*	-	0.758	1.263	0.753	0.220	1.286	0.073	0.740
*P*	-	0.470	0.532	0.472	0.802	0.278	0.930	0.478
TT	183	4.35 ± 1.14	1.03(0.74)	1.69 ± 0.48	2.54 ± 0.96	1.35 ± 0.37	0.81 ± 0.23	1.83 ± 0.91
TC/CC	105	4.41 ± 0.91	1.23(1.08)	1.74 ± 0.46	2.50 ± 0.82	1.39 ± 0.34	0.82 ± 0.24	1.87 ± 0.89
*F*	-	0.311	0.850	0.864	0.091	0.821	0.146	0.166
*P*	-	0.578	0.395	0.353	0.763	0.366	0.703	0.684
Female								
TT	186	4.36 ± 0.79	0.94(0.51)	1.66 ± 0.35	2.64 ± 0.63	1.28 ± 0.26	0.86 ± 0.20	1.55 ± 0.46
TC	113	4.23 ± 0.69	0.92(0.56)	1.67 ± 0.32	2.50 ± 0.57	1.27 ± 0.22	0.82 ± 0.24	1.66 ± 0.53
CC	22	4.57 ± 0.92	0.81(0.45)	1.65 ± 0.52	2.87 ± 0.74	1.27 ± 0.34	0.93 ± 0.21	1.41 ± 0.44
*F*	-	2.265	0.697	0.031	3.759	0.064	3.264	3.280
*P*	-	0.106	0.706	0.969	0.024	0.938	0.040	0.039
TT	186	4.36 ± 0.79	0.94(0.51)	1.66 ± 0.35	2.64 ± 0.63	1.28 ± 0.26	0.86 ± 0.20	1.55 ± 0.46
TC/CC	135	4.28 ± 0.74	0.91(0.54)	1.67 ± 0.36	2.57 ± 0.61	1.27 ± 0.24	0.84 ± 0.24	1.62 ± 0.52
*F*	-	0.795	0.799	0.030	1.086	0.129	1.289	1.565
*P*	-	0.373	0.425	0.862	0.298	0.720	0.257	0.212
Han Chinese								
TT	299	4.65 ± 0.91	0.99(0.39)	1.92 ± 0.48	2.56 ± 0.67	1.40 ± 0.24	0.88 ± 0.20	1.66 ± 0.43
TC	232	4.87 ± 1.14	1.02(0.80)	1.93 ± 0.51	2.64 ± 0.81	1.42 ± 0.25	0.94 ± 0.23	1.61 ± 0.46
CC	42	4.90 ± 1.19	1.18(0.68)	1.91 ± 0.50	2.78 ± 1.01	1.42 ± 0.22	0.95 ± 0.25	1.67 ± 0.94
*F*	-	3.194	2.428	0.041	1.964	0.449	4.601	0.742
*P*	-	0.042	0.297	0.959	0.141	0.639	0.010	0.477
TT	299	4.65 ± 0.91	0.99(0.39)	1.92 ± 0.48	2.56 ± 0.67	1.40 ± 0.24	0.88 ± 0.20	1.66 ± 0.43
TC/CC	274	4.87 ± 1.14	1.04(0.77)	1.93 ± 0.51	2.66 ± 0.84	1.42 ± 0.25	0.94 ± 0.24	1.62 ± 0.56
*F*	-	6.354	1.137	0.048	2.699	0.899	9.048	0.985
*P*	-	0.012	0.255	0.827	0.101	0.344	0.003	0.321
Male								
TT	146	4.59 ± 0.96	1.01(0.63)	1.87 ± 0.53	2.51 ± 0.64	1.39 ± 0.28	0.88 ± 0.19	1.63 ± 0.39
TC	97	5.01 ± 1.34	1.14(1.08)	1.85 ± 0.58	2.66 ± 0.91	1.38 ± 0.27	0.93 ± 0.24	1.58 ± 0.47
CC	17	4.78 ± 0.76	1.15(1.07)	1.93 ± 0.60	2.56 ± 0.43	1.40 ± 0.07	0.89 ± 0.24	1.84 ± 1.33
*F*	-	4.160	2.542	0.164	1.087	0.027	1.821	1.901
*P*	-	0.017	0.281	0.849	0.339	0.973	0.164	0.152
TT	146	4.59 ± 0.96	1.01(0.63)	1.87 ± 0.53	2.51 ± 0.64	1.39 ± 0.28	0.88 ± 0.19	1.63 ± 0.39
TC/CC	114	4.98 ± 1.27	1.14(1.02)	1.86 ± 0.55	2.64 ± 0.85	1.39 ± 0.26	0.93 ± 0.24	1.62 ± 0.67
*F*	-	7.690	1.591	0.016	1.940	0.004	3.205	0.070
*P*	-	0.006	0.112	0.900	0.165	0.951	0.075	0.792
Female								
TT	153	4.71 ± 0.85	0.97(0.56)	1.96 ± 0.44	2.60 ± 0.70	1.42 ± 0.20	0.89 ± 0.21	1.68 ± 0.46
TC	135	4.76 ± 0.95	0.95(0.68)	1.98 ± 0.45	2.63 ± 0.73	1.45 ± 0.23	0.94 ± 0.23	1.63 ± 0.45
CC	25	4.98 ± 1.43	1.19(0.57)	1.90 ± 0.44	2.93 ± 1.25	1.44 ± 0.25	0.99 ± 0.26	1.54 ± 0.52
*F*	-	0.887	2.362	0.406	2.009	0.822	3.265	1.120
*P*	-	0.413	0.307	0.667	0.136	0.441	0.039	0.328
TT	153	4.71 ± 0.85	0.97(0.56)	1.96 ± 0.44	2.60 ± 0.70	1.42 ± 0.20	0.89 ± 0.21	1.68 ± 0.46
TC/CC	160	4.73 ± 1.04	0.99(0.65)	1.97 ± 0.45	2.67 ± 0.83	1.45 ± 0.23	0.94 ± 0.23	1.61 ± 0.46
*F*	-	0.584	0.196	0.046	0.757	1.555	5.419	1.493
*P*	-	0.445	0.844	0.830	0.385	0.213	0.021	0.223

TC, total cholesterol; TG, triglycerides; HDL-C, high-density lipoprotein cholesterol; LDL-C, low-density lipoprotein cholesterol; ApoAI, apolipoprotein AI; ApoB, apolipoprotein B; ApoAI/ApoB, the ratio of apolipoprotein AI to apolipoprotein B. The value of TG was presented as median (interquartile range). The difference among the genotypes was determined by the Kruskal-Wallis test or the Wilcoxon-Mann-Whitney test.

The levels of TC and ApoB in the total Han population were significant difference among the three genotypes (*P *< 0.05 for all). The C allele carriers (TC and CC genotypes) had higher serum TC and ApoB levels as compared with the C allele noncarriers (TT genotype). When serum lipid levels were analyzed according to sex, the difference in serum TC levels in Han Chinese was significant in males (*P *< 0.01) but not in females, whereas the difference in serum ApoB levels was significant in females (*P *< 0.05) but not in males.

There was no significant difference in the remaining serum lipid parameters among the three genotypes in Bai Ku Yao, Han, males, or females (*P *> 0.05 for all); respectively.

### Relative factors for serum lipid parameters

Multiple linear regression analysis showed that serum TC, TG and ApoB levels were correlated with genotypes in the combined population of Bai Ku Yao and Han (*P *< 0.05 for all). Serum TC and ApoB levels were correlated with genotypes in Han (*P *< 0.05 for each) but not in Bai Ku Yao (Table 4). Serum lipid parameters were also correlated with sex, age, BMI, alcohol consumption, cigarette smoking, and blood pressure in both ethnic groups (Table 4).

**Table 4:** Correlative factors for serum lipid parameters between Bai Ku Yao and Han Chinese

Lipid parameter	Risk factor	Unstandardized coefficient	Std. error	Standardized coefficient	*t*	*P*
Yao plus Han						
TC	Body mass index	0.065	0.010	0.177	6.332	0.000
	Ethnic group	0.373	0.055	0.187	6.810	0.000
	Age	0.009	0.002	0.140	4.926	0.000
	Diastolic blood pressure	0.009	0.003	0.094	3.248	0.001
	Genotype	0.087	0.045	0.054	1.963	0.050
TG	Body mass index	0.097	0.014	0.195	6.963	0.000
	Alcohol consumption	0.174	0.042	0.131	4.122	0.000
	Sex	-0.257	0.087	-0. 094	-2.967	0.003
	Genotype	0.123	0.062	0.056	1.980	0.048
HDL-C	Ethnic group	0.250	0.025	0.266	9.840	0.000
	Age	0.006	0.001	0.191	6.979	0.000
	Alcohol consumption	0.086	0.014	0.187	6.035	0.000
	Sex	0.111	0.029	0.117	3.846	0.000
	Body mass index	-0.017	0.005	-0.102	-3.763	0.000
LDL-C	Body mass index	0.054	0.008	0.194	6.870	0.000
	Age	0.007	0.001	0.135	4.724	0.000
	Alcohol consumption	-0.087	0.021	-0.116	-4.055	0.000
ApoAI	Alcohol consumption	0.067	0.009	0.240	7.605	0.000
	Ethnic group	0.101	0.016	0.177	6.432	0.000
	Age	0.003	0.001	0.165	5.903	0.000
	Sex	0.040	0.018	0.070	2.235	0.026
ApoB	Body mass index	0.017	0.002	0.200	7.125	0.000
	Age	0.003	0.000	0.191	6.656	0.000
	Ethnic group	0.066	0.013	0.145	5.284	0.000
	Alcohol consumption	-0.019	0.006	-0.087	-3.078	0.002
	Diastolic blood pressure	0.001	0.001	0.068	2.310	0.021
	Genotype	0.023	0.010	0.065	2.234	0.026
ApoAI/ApoB	Alcohol consumption	0.133	0.017	0.218	7.608	0.000
	Body mass index	-0.034	0.006	-0.148	-5.250	0.000
	Age	-0.002	0.001	-0.062	-2.169	0.030
Bai Ku Yao						
TC	Body mass index	0.072	0.015	0.189	4.776	0.000
	Age	0.008	0.002	0.142	3.598	0.000
TG	Alcohol consumption	0.094	0.040	0.110	2.329	0.020
	Body mass index	0.054	0.015	0.139	3.503	0.000
	Sex	-0.238	0.087	-0.128	-2.735	0.006
HDL-C	Alcohol consumption	0.069	0.015	0.180	4.547	0.000
	Age	0.005	0.001	0.171	4.335	0.000
LDL-C	Body mass index	0.063	0.013	0.196	4.954	0.000
	Age	0.006	0.002	0.129	3.245	0.001
	Cigarette smoking	-0.062	0.025	-0.097	-2.446	0.015
ApoAI	Alcohol consumption	0.082	0.011	0.283	7.324	0.000
	Age	0.003	0.001	0.136	3.523	0.000
ApoB	Body mass index	0.020	0.004	0.208	5.286	0.000
	Age	0.002	0.001	0.167	4.209	0.000
	Alcohol consumption	-0.023	0.008	-0.109	-2.753	0.006
ApoAI/ApoB	Alcohol consumption	0.197	0.026	0.295	7.605	0.000
Han Chinese						
TC	Diastolic blood pressure	0.013	0.004	0.135	3.235	0.001
	Body mass index	0.060	0.014	0.176	4.350	0.000
	Age	0.010	0.003	0.163	3.964	0.000
	Genotype	0.136	0.065	0.083	2.081	0.038
TG	Body mass index	0.127	0.022	0.226	5.648	0.000
	Alcohol consumption	0.353	0.071	0.198	4.954	0.000
HDL-C	Age	0.008	0.001	0.242	5.975	0.000
	Body mass index	-0.023	0.006	-0.140	-3.557	0.000
	Sex	0.158	0.042	0.160	3.760	0.000
	Alcohol consumption	0.072	0.022	0.140	3.211	0.001
LDL-C	Body mass index	0.046	0.010	0.185	4.584	0.000
	Alcohol consumption	-0.119	0.033	-0.150	-3.610	0.000
	Age	0.007	0.002	0.148	3.575	0.000
ApoAI	Age	0.004	0.001	0.232	5.694	0.000
	Sex	0.082	0.021	0.168	3.917	0.000
	Alcohol consumption	0.039	0.011	0.155	3.527	0.000
ApoB	Age	0.003	0.001	0.239	6.076	0.000
	Body mass index	0.016	0.003	0.218	5.540	0.000
	Genotype	0.035	0.014	0.098	2.498	0.013
ApoAI/ApoB	Body mass index	-0.032	0.007	-0.197	-4.794	0.000

TC, total cholesterol; TG, triglyceride; HDL-C, high-density lipoprotein cholesterol; LDL-C, low-density lipoprotein cholesterol; ApoAI, apolipoprotein AI; ApoB, apolipoprotein B.

## Discussion

The present study showed that the levels of serum TC, HDL-C, ApoAI and ApoB were lower in Bai Ku Yao than in Han Chinese. There was no significant difference in the serum levels of TG, LDL-C and the ratio of ApoAI to ApoB between the two ethnic groups. As aforementioned, dyslipidemia is a multifactorial origin, including hereditary and acquired risk factors and their interactions [11,12]. Bai Ku Yao is a special and isolated subgroup of the Yao minority in China. They reside in two villages, Lihu and Baxu, Nandan County. Both Lihu and Baxu villages are typical infertile mountain region, usually it was called 30 percent soil with 70 percent rock. Thus, their income mostly comes from planting corn and paddy. Strict intra-ethnic marriages have been performed in this ethnic subgroup from time immemorial. Therefore, we believe that the variation of some lipid metabolism-related genes in this population may be different from those in Han Chinese.

The genotypic and allelic distribution of PPARD +294T > C polymorphism was different in diverse populations. Several previous studies have showed that the frequency of the rare allele (PPARD +294C) was significantly higher in Russian endurance-oriented athletes than in controls (18.3% vs. 12.1%, *P *< 0.0001) [42], in Tunisian CAD patients than in healthy volunteers (32.0% vs. 18.9%, *P *= 0.001) [34], and in Chinese CAD patients than in normal controls (30.8% vs. 19.5%, *P *< 0.05) [35]. Some studies, however, showed that there was no difference in its frequency between the patients with diabetes mellitus type 2 and the non-diabetic controls (18.7% versus 19.2%, *P *> 0.999) [37], or among the patients with metabolic syndrome, essential hypertension and diabetes mellitus type 2 [30]. In the present study, we showed that the frequency of PPARD +294C allele was lower in Bai Ku Yao (22.50%) than in Han Chinese (27.57%). The frequency of TC and CC genotypes was also lower in Bai Ku Yao than in Han. However, the frequency of PPARD +294C allele was higher in both ethnic groups than in 543 healthy 50-year-old-men (15.6%) from the northern part of the greater Stockholm area [28], in normal controls (19.5%) from Chinese Anhui Province [35], in healthy Tunisian population (18.9%) [34], and in non-diabetic Germany controls (19.2%) [37]; but it was lower than in Tunisian CAD patients (32.0%) [34] and Chinese CAD patients (30.8%) [35]. These results indicate that the prevalence of the C allele variants of PPARD +294T > C polymorphism may have an ethnic specificity.

The association of PPARD +294T > C polymorphism and plasma or serum lipid levels in humans has been evaluated in several previous studies. However, the findings are inconsistent. Skogsberg *et al*. [28] demonstrated that this SNP was implicated in cholesterol metabolism in Swedish men. Homozygotes for the rare C allele had a higher plasma LDL-C concentration than homozygotes for the common T allele, which was verified in an independent cohort consisting of 282 healthy men, while there were no associations with the HDL-C levels. Interestingly, the same group of investigators showed in another study in Scottish men that the SNP did not influence LDL-C concentrations but was associated with lower HDL-C levels. Individuals carrying the rare C allele had a significantly lower HDL-C concentration than subjects homozygous for the common T allele [29]. Moreover, Aberle *et al*. [31] showed a highly significant association between the rare C allele and lower plasma HDL-C concentrations in dyslipidemic female subjects. The effect remained significant after correcting for multiparametric testing according to Bonferoni and was seen only in subjects with a BMI below the median. In addition, several recent reports have also uniformly showed that the PPARD +294T > C polymorphism was associated with some serum lipid phenotypes. For example, metabolic syndrome patients with CC genotype had significantly higher TC and LDL-C levels than those with TT and TC genotypes [30]. Among subjects with and without type 2 diabetes, the PPARD +294T > C polymorphism was associated with HDL-C and was dependent on sex [32]. The risk variant of PPARD +294T > C marker was associated with higher LDL-C and increased serum TC [33]. However, Gouni-Berthold *et al*. [37] found that the presence of the C allele had no effect on TG, HDL-C, and LDL-C levels, both in diabetic and non-diabetic German controls, or both in men and in women. The same result was found by Jguirim-Souissi *et al*. [34] both in CAD patients and healthy controls. In the present study, we showed that the levels of LDL-C, ApoB and the ratio of ApoAI to ApoB in Bai Ku Yao were significant difference among the three genotypes in females but not in males. The subjects with CC genotype in females had higher serum LDL-C and ApoB levels and lower the ratio of ApoAI to ApoB than the subjects with TT and TC genotypes. In contrast, the levels of TC and ApoB in the total Han population were significant difference among the three genotypes. The C allele carriers had higher serum TC and ApoB levels as compared with the C allele noncarriers. When serum lipid levels were analyzed according to sex, the difference in serum TC levels in Han Chinese was significant in males but not in females, whereas the difference in serum ApoB levels was significant in females but not in males. Serum TC and ApoB levels were correlated with genotypes in Han but not in Bai Ku Yao. These results suggest that the association of PPARD +294T > C polymorphism and serum lipid levels is different between the Bai Ku Yao and Han populations. The discrepancy between the two ethnic groups might partly result from different PPARD +294T > C polymorphism or PPARD gene-enviromental interactions.

In the present study, we also found that serum lipid parameters were associated with age, sex, alcohol consumption, cigarette smoking, BMI, and blood pressure. These results suggest that the environmental factors also play important roles in dyslipidemia in our populations [6,7]. The dietary patterns and lifestyle between the two ethnic groups were different. The staple food was corn and the subsidiary foods were rice, soy, buckwheat, sweet potato, and pumpkin products in Bai Ku Yao. Approximately 90% of the beverages were corn wine and rum. The alcohol content is about 15% (v/v). The people of Bai Ku Yao are also accustomed to drink hempseed soup and eat hempseed products. In contrast, rice was the staple food and corn, broomcorn, potato, and taro products were the subsidiary foods in Han. About 90% of the beverage was rice wine. The content of alcohol is about 30% (v/v). The staple and subsidiary foods are more favorable for serum lipid profiles in Bai Ku Yao than in Han. Corn contains abundant dietary fiber and plant protein [43]. Consumption of dietary fiber can decrease serum lipid levels [44,45]. A dietary supplement of water-soluble fibers (guar gum, pectin) and mostly non-water-soluble fibers (soy fiber, pea fiber, corn bran) in subjects with mild to moderate hypercholesterolemia (LDL-C, 3.37-4.92 mmol/L) had significant blood cholesterol-lowering effects. The mean decreases during the 15-week period for LDL-C, TC, and LDL-C/HDL-C ratio were greater (*P *< 0.001) in the fiber group. The mean changes from pre-treatment values in LDL-C, TC, and LDL-C/HDL-C ratio for subjects in the fiber group were -0.51 mmol/L (-12.1%), -0.53 mmol/L (-8.5%), and -0.30 (-9.4%); respectively. But the fiber supplement had no significant effects on HDL-C and TG [45]. Plant protein might promote the transportation and excretion of free cholesterol. Dietary soy protein has well-documented beneficial effects on serum lipid concentrations [46,47]. Soy protein with isoflavones intact was associated with significant decreases in serum TC (by 0.22 mmol/L, or 3.77%), LDL-C (by 0.21 mmol/L, or 5.25%), and TG (by 0.10 mmol/L, or 7.27%) and significant increases in serum HDL-C (by 0.04 mmol/L, or 3.03%). The reductions in total and LDL-C were larger in men than in women. Initial TC concentrations had a powerful effect on changes in total and HDL-C, especially in subjects with hypercholesterolemia. Studies with intakes > 80 mg showed better effects on the lipid profile. The strongest lowering effects of soy protein containing isoflavones on TC, LDL-C, and TG occurred within the short initial period of intervention, whereas improvements in HDL-C were only observed in studies of > 12 wk duration [47]. Buckwheat protein product has a potent hypocholesterolemic activity [48,49]. Plasma TC concentrations in rats fed a cholesterol-free diet with tartary buckwheat sprout powder were significantly lower than in the control rats fed a diet with alpha-cornstarch. The cholesterol-lowering function of tartary buckwheat sprout powder may be achieved by enhancing fecal bile acid excretion through increased fecal matter excretion or the upregulation of hepatic cholesterol 7alpha-hydroxylase mRNA expression [49]. Ingestion of 4 g/day caiapo (the extract of the white-skinned sweet potato Ipomoea batatas) for 6 weeks has been found to reduce plasma TC and LDL-C levels in type 2 diabetic patients previously treated by diet alone [50]. Chang *et al*. [51] have evaluated the effect of purple sweet potato leaves (PSPLs) consumption on antioxidative status and its modulation of that status in basketball players during training period. The results showed that LDL lag time was significantly longer in the PSPLs group, sugesting that consumption of PSPLs diet for 2 weeks may reduce lipid and DNA oxidation that can modulate the antioxidative status of basketball players during training period. Adaramoye *et al*. [52] reported that supplemented diets containing 3% and 6% telfairia occidentalis (fluted pumpkin) in rats decreased plasma and postmitochondrial supernatant fraction (PMF) cholesterol levels by 20% and 30% and by 30% and 45%, respectively; decreased the cholesterol-induced increase in plasma and PMF LDL-C levels by 24% and 48% and by 28% and 52%, respectively; and decreased plasma and PMF lipid peroxidation by 24% and 20% and by 42% and 21%, respectively. Hempseed contains a high proportion of the polyunsaturated fatty acids (PUFAs) such as linoleic acid, linolenic acid, oleic acid, palmitic acid, and stearic acid, which may have beneficial effects on serum lipid profiles. Hempseed-supplemented diet in animals displayed elevated plasma levels of PUFAs and a prominent enhancement in gamma-linolenic acid levels. When hempseed is added to a cholesterol-enriched diet, cholesterol-induced platelet aggregation returns to control levels [53,54]. This normalization may be partly due to increased levels of plasma gamma-linolenic acid [53]. In addition, several experimental and clinical studies have demonstrated that dietary hempseed or hempseed oil can decrease serum TC, TG and LDL-C levels [55-57], inhibit lipid peroxidation [58], and reduce atherogenic index [59].

## Conclusion

The present study shows that the frequency of PPARD +294C allele was lower in Bai Ku Yao than in Han Chinese. The subjects with CC genotype in Bai Ku Yao had higher serum LDL-C and ApoB levels and lower the ratio of ApoAI to ApoB than the subjects with TT and TC genotypes in females but not in males. The C allele carriers in Han Chinese had higher serum TC and ApoB levels than the C allele noncarriers. When serum lipid levels were analyzed according to sex, the difference in serum TC levels in Han Chinese was significant in males but not in females, whereas the difference in serum ApoB levels was significant in females but not in males. Serum TC and ApoB levels were correlated with genotypes in Han but not in Bai Ku Yao. These results suggest that the association of PPARD +294T > C polymorphism and serum lipid levels is different between the Bai Ku Yao and Han populations. The difference in the association of PPARD +294T > C polymorphism and serum lipid levels between the two ethnic groups might partly result from different PPARD +294T > C polymorphism or PPARD gene-enviromental interactions.

## Competing interests

The authors declare that they have no competing interests.

## Authors' contributions

LM participated in the design, undertook genotyping, and helped to draft the manuscript. RXY conceived the study, participated in the design, carried out the epidemiological survey, collected the samples, and drafted the manuscript. DFW, XLC, QL, XJH, TTY and LHHA collaborated to the genotyping. DZY and WXL carried out the epidemiological survey, collected the samples, and helped to carry out the genotyping. All authors read and approved the final manuscript.
